# Exogenous Sodium Nitroprusside Affects the Redox System of Wheat Roots Differentially Regulating the Activity of Antioxidant Enzymes under Short-Time Osmotic Stress

**DOI:** 10.3390/plants13141895

**Published:** 2024-07-09

**Authors:** Alsu Lubyanova, Chulpan Allagulova

**Affiliations:** Institute of Biochemistry and Genetics-Subdivision of the Ufa Federal Research Centre of the Russian Academy of Sciences, Prospect Oktyabrya 71, lit.1e, 450054 Ufa, Russia; allagulova-chulpan@rambler.ru

**Keywords:** catalase, electrolyte leakage, hydrogen peroxide, malondialdehyde, ascorbate peroxidase, polyethylene glycol, proline, superoxide dismutase, superoxide anion, *Triticum aestivum* L.

## Abstract

Nitric oxide (NO) is a multifunctional signalling molecule involved in the regulation of plant ontogenesis and adaptation to different adverse environmental factors, in particular to osmotic stress. Understanding NO-induced plant protection is important for the improvement of plant stress tolerance and crop productivity under global climate changes. The root system is crucial for plant survival in a changeable environment. Damages that it experiences under water deficit conditions during the initial developmental periods seriously affect the viability of the plants. This work was devoted to the comparative analysis of the pretreatment of wheat seedlings through the root system with NO donor sodium nitroprusside (SNP) for 24 h on various parameters of redox homeostasis under exposure to osmotic stress (PEG 6000, 12%) over 0.5–24 h. The active and exhausted solutions of SNP, termed as (SNP/+NO) and (SNP/−NO), respectively, were used in this work at a concentration of 2 × 10^−4^ M. Using biochemistry and light microscopy methods, it has been revealed that osmotic stress caused oxidative damages and the disruption of membrane cell structures in wheat roots. PEG exposure increased the production of superoxide (O_2_^•−^), hydrogen peroxide (H_2_O_2_), malondialdehyde (MDA), and the levels of electrolyte leakage (EL) and lipid peroxidation (LPO). Stress treatment enhanced the activities of superoxide dismutase (SOD), ascorbate peroxidase (APX), catalase (CAT), the excretion of proline, and the rate of cell death and inhibited their division. Pretreatment with (SNP/+NO) decreased PEG-induced root damages by differently regulating the antioxidant enzymes under stress conditions. Thus, (SNP/+NO) pretreatment led to SOD, APX, and CAT inhibition during the first 4 h of stress and stimulated their activity after 24 h of PEG exposure when compared to SNP-untreated or (SNP/−NO)-pretreated and stress-subjected plants. Osmotic stress triggered the intense excretion of proline by roots into the external medium. Pretreatment with (SNP/+NO) in contrast with (SNP/−NO) additionally increased stress-induced proline excretion. Our results indicate that NO is able to mitigate the destructive effects of osmotic stress on the roots of wheat seedlings. However, the mechanisms of NO protective action may be different at certain periods of stress exposure.

## 1. Introduction

Plants are periodically affected by unfavourable environmental factors, which have recently increased significantly due to global climate change and human economic activity. Drought, salinity, extreme temperatures, excess of fertilizers, and heavy metals are the most widespread negative factors, all leading to water regime disturbances initiating osmotic and oxidative stress. Water deficit becomes especially damaging in early ontogeny, during germination and seedling emergence, inhibiting further plant growth and ultimately crop productivity. The root system is crucial for plant survival in a changeable environment, providing water and nutrient uptake, anchorage to the substrate, and signal transduction to the shoots or surrounding medium [1]. Damages to a root system under water deficit conditions at the initial periods of vegetation can seriously affect plant viability, increasing the death rate dramatically. The reactions of roots to external factors may be different than in aerial parts of the plants [2,3,4,5]. The information related to the cellular and molecular mechanisms of roots development and their stress tolerance is mainly based on the studies of dicot plant species. However, many crops, including wheat (*Triticum aestivum* L.), which is the world’s major cereal, are classified as monocots. Therefore, the study of root reactions in wheat plants at the early stages of development under osmotic stress has a fundamental importance.

There are different adaptive mechanisms of plant survival under water deficit, among which the most common one is osmotic adjustment, which involves a diversity of osmoprotective compounds such as polyamines, sugars, and amino acids, including proline (Pro) [6]. Proline also has antioxidant properties, contributing to plant defence reactions against the damaging effects of reactive oxygen species (ROS) such as superoxide anion radical (O_2_^•−^), hydrogen peroxide (H_2_O_2_), hydroxyl radicals (^•^OH), and singlet oxygen (^1^O_2_) [7,8]. Stress-induced oxidative burst leads to the injury of biomolecules including nucleic acids, lipids, or proteins, causing the degradation of cellular structures [9,10]. Membrane damages are coupled with an increase in malondialdehyde (MDA) accumulation and enhanced electrolyte leakage (EL). ROS neutralization and the maintenance of redox homeostasis occur due the action of the plant’s antioxidant system (AOS), composed of non-enzymatic components and antioxidant enzymes like superoxide dismutases (SODs), catalases (CATs), and peroxidases (PODs), in particular, ascorbate peroxidase (APX), etc. SOD catalyses the transformation of toxic O_2_^•−^ to less toxic H_2_O_2_, which is ultimately converted to O_2_ and H_2_O through POD or CAT activity [9,11,12].

Plant defence reactions and the establishment of their stress resistance are regulated by a complex signalling system through interaction with molecular messengers, among which nitric oxide (NO) has received significant attention [10]. NO is a gaseous redox-active signalling molecule which fulfils multiple regulatory functions at all stages of the plant life cycle, including seed germination, vegetative growth, root morphogenesis, flowering, and fruit ripening [13,14]. It plays an important role in the adaptation of plants to numerous stresses, in particular, water deficit. The stress-induced increase in endogenous NO levels is well known, as is the enhancement of plant stress tolerance by the exogenous application of NO in gaseous form or as donors [15]. However, excessive amounts are toxic and can induce nitro-oxidative stress in tissues [13,16]. Therefore, practical use requires scrupulous study regarding the mechanisms of NO action and those of its active derivatives—reactive nitrogen species (RNS). Their biological activity is realized through interaction with various mediators (glutathione, ROS, polyunsaturated fatty acids), with subsequent signal transduction to its own molecular targets [10,14]. For example, when NO interacts with O_2_^•−^, peroxynitrite (NOOO^−^) is formed, which is characterized by less toxic properties than O_2_^•−^. Thus, NOOO^−^ formation can be recognized as a direct antioxidant effect of NO [10]. In addition, NOOO^−^ acts as a nitrating agent in reactions of tyrosine nitration in NO-dependent post-translational modifications of proteins (NO-PTMs). Besides tyrosine nitration, other types of NO-PTMs are S-nitrosylation and metal nitrosylation, all leading to the structural reorganization of target proteins, thus changing their functional activity [14]. For instance, NO-induced S-nitrosylation of APX1 at cysteine-32 enhances its enzymatic activity in *Arabidopsis* [17]. Potential targets of S-nitrosylation are the protein molecules SOD and CAT, so their enzymatic activity can be regulated by NO-PTMs [18,19,20], which could contribute significantly to the NO-dependent regulation of the redox homeostasis of plants.

Experimental studies of NO functional activity are commonly conducted using different donors, in particular, sodium nitroprusside (SNP), which is an unstable photosensitive compound with a half-life time of about 12 h [10,13]. Therefore, the assessment of its effects must be performed with parallel additional control, such as exhausted SNP [21]. This work was devoted to a comparative analysis of the effects of active and exhausted SNP, termed as (SNP/+NO) and (SNP/−NO), respectively [21], on various parameters of redox homeostasis in roots of wheat seedlings subjected to osmotic stress. O_2_^•−^, H_2_O_2_ production, antioxidant enzyme activity (APX, SOD, and CAT), MDA accumulation, EL, the level of cell viability, mitotic activity, and the integrity of cellular structures were quantified in the roots of SNP-pretreated and stress-subjected seedlings. 

## 2. Results

### 2.1. O_2_^•−^ Production and SOD Activity in Roots of Wheat Seedlings Pretreated with SNP and Subjected to Osmotic Stress for 4 h

**Figure 1:**
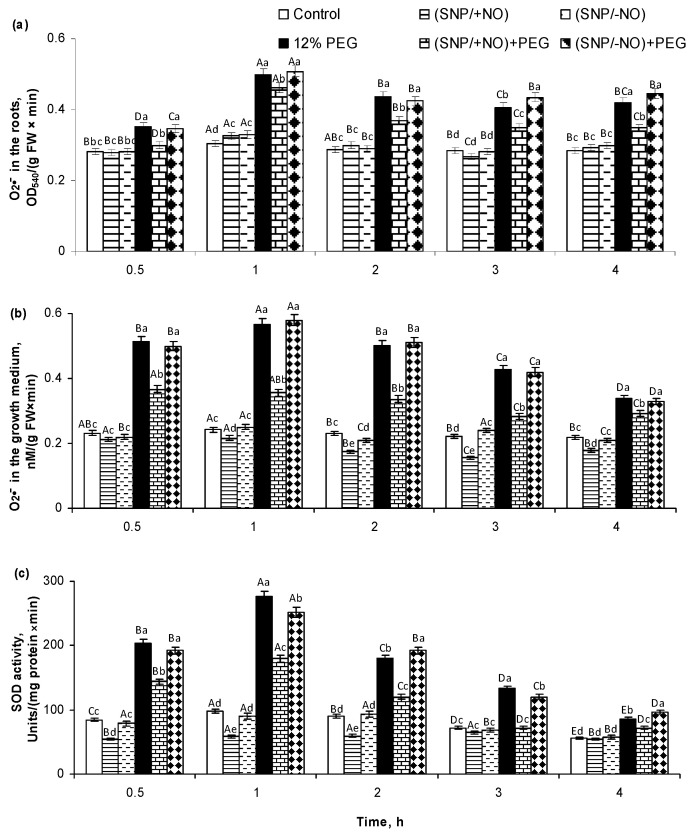
Effect of 2 × 10^−4^ M sodium nitroprusside (SNP) pretreatment of 5-day-old wheat seedlings for 24 h on superoxide (O_2_^•−^) production in roots (**a**), O_2_^•−^ accumulation in the growth medium (**b**), and superoxide dismutase (SOD) activity in the roots (**c**) of 6-day-old seedlings subjected to short-term (4 h) osmotic stress, caused by 12% polyethylene glycol 6000 (PEG). (SNP/+NO)—active SNP; (SNP/−NO)—exhausted (light-inactivated) SNP. Data are given as mean values and their standard errors from three biological and four analytical repeats. Lowercase letters above the columns indicate significant differences between variants of treatment at the particular time point at *p* < 0.05 (ANOVA, LSD test). Capital letters indicate significant differences for the same treatment at different time points at *p* < 0.05 (ANOVA, LSD test).

The production of O_2_^•−^ by roots was evaluated from its accumulation both in the roots and in the growth medium of SNP-pretreated and osmotic-stress-subjected wheat seedlings. The (SNP/+NO) pretreatment of 5-day-old seedlings had no effect on O_2_^•−^ accumulation in roots and reduced its level in the growth medium by 8–29% relative to the control (Figure 1a,b). Osmotic stress induced an enhancement of O_2_^•−^ levels in the root tissues as well as in the growth medium. O_2_^•−^ content increased in roots by 26–67% relative to the control, with a maximum observed at 1 h of stress exposure (Figure 1a). The most pronounced increase in O_2_^•−^ accumulation in the growth medium, exceeding the control by more than two-fold, was also observed after 1 h of stress treatment (Figure 1b). Then, the content of O_2_^•−^ in the medium gradually decreased but still elevated in the control in the next 2–4 h of the experiment (Figure 1b). (SNP/+NO) pretreatment decreased, but did not prevent, stress-induced O_2_^•−^ production by seedlings. The levels of O_2_^•−^ accumulation under (SNP/+NO) pretreatment followed by 4 h of stress exposure exceeded the control by 7–53% in the roots and by 30–60% in the growth medium.

SOD activity in wheat roots after the first 2 h of (SNP/+NO) pretreatment was 33–40% lower than the control, which then gradually increased, reaching control values at 4 h (Figure 1c). Stress treatment caused a sharp increase in this enzyme’s activity. A maximum 2.4- to 2.8-fold stimulation of SOD activity was observed in roots after 0.5–1 h of stress exposure, which then gradually decreased. (SNP/+NO) pretreatment decreased the level of stress-induced enzyme activation. In this case, the roots of (SNP/+NO)-pretreated and stress-subjected seedlings differed from control by just a 1.7- to 1.9-fold increase in the SOD activity. No changes were detected in the roots and in the medium for O_2_^•−^ accumulation and also for root’s SOD activity under pretreatment with a 2 × 10^−4^ M solution of exhausted NO donor (SNP/−NO) both in normal and stressful conditions.

### 2.2. H_2_O_2_ Production and Activity of APX and CAT in Roots of Wheat Seedlings Pretreated with SNP and Subjected to Osmotic Stress for 4 h

**Figure 2:**
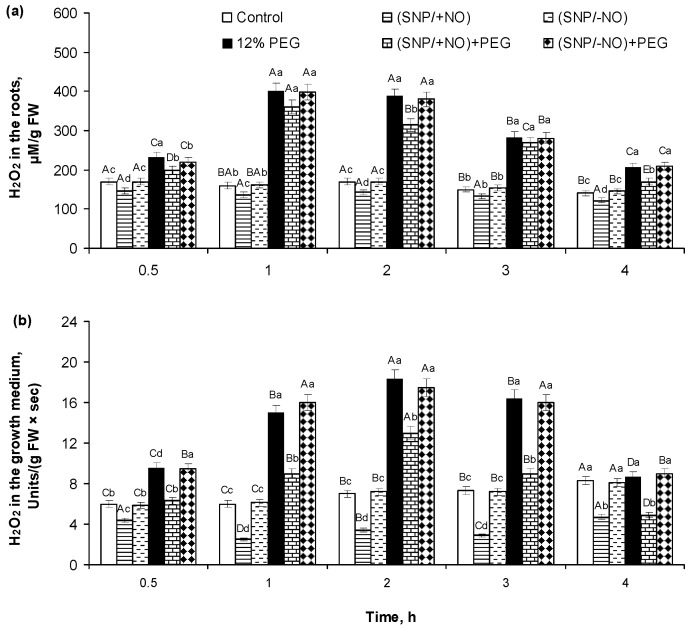
Effect of SNP pretreatment of wheat seedlings on hydrogen peroxide (H_2_O_2_) accumulation in roots (**a**) and in the growth medium (**b**) of 6-day-old seedlings subjected to short-term (0.5–4 h) osmotic stress, caused by PEG exposure. Data are given as mean values and their standard errors from three experimental and four analytical repeats. Lowercase letters above the columns indicate significant differences between variants of treatment at the particular time point at *p* < 0.05 (ANOVA, LSD test). Capital letters indicate significant differences for the same treatment at different time points at *p* < 0.05 (ANOVA, LSD test).

The production of hydrogen peroxide by roots was also analysed by its endogenous content in roots and accumulation in the external medium of SNP-pretreated seedlings subjected to PEG-induced osmotic stress, and it had a similar trend with O_2_^•−^ (Figure 2).

(SNP/+NO) pretreatment reduced root endogenous levels of H_2_O_2_ by 10–15% (Figure 2a) and its excretion to the medium by 30–60% (Figure 2b) as compared to control. Osmotic stress resulted in a rapid 1.5–2.5-fold increase in H_2_O_2_ content in roots and in the growth medium during the first 4 h of PEG exposure. The maximum H_2_O_2_ accumulation occurred at 1 h of 12% PEG influence. (SNP/+NO) pretreatment decreased the stress-induced accumulation of H_2_O_2_ both in the roots and in the growth medium. H_2_O_2_ content increased 1.2–2.3-fold in the root tissues and 1.1–1.9-fold in the growth medium over the control level. The maximum levels of H_2_O_2_ accumulation were detected in roots after 1–3 h and in the growth medium after 1–2 h of stress influence. The (SNP/-NO) pretreatment showed no effect of active SNP on the root H_2_O_2_ production and excretion under 12% PEG exposure (Figure 2a,b).

**Figure 3:**
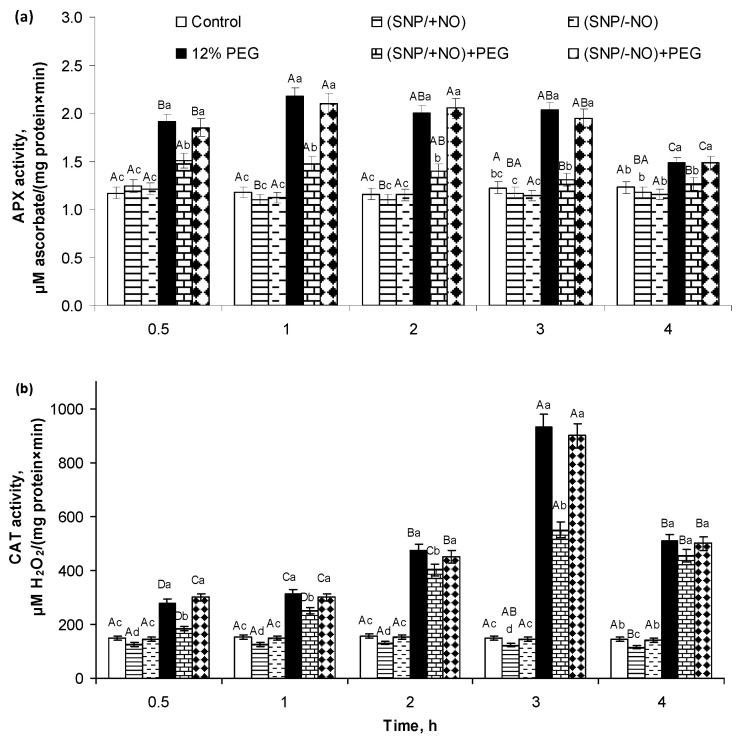
Effect of SNP pretreatment of wheat seedlings on activities of ascorbate peroxidase (APX) (**a**) and catalase (CAT) (**b**) in roots of 6-day-old seedlings subjected to short-term (0.5–4 h) osmotic stress, caused by 12% PEG exposure. Data are given as mean values and their standard errors from three experimental and four analytical repeats. Lowercase letters above the columns indicate significant differences between variants of treatment at the particular time point at *p* < 0.05 (ANOVA, LSD test). Capital letters indicate significant differences for the same treatment at different time points at *p* < 0.05 (ANOVA, LSD test).

Roots of (SNP/+NO)-pretreated seedlings were characterized by no significant changes in APX activity and by 17–23% reduction in CAT activity as compared to control (Figure 3). The activity of these related to the regulation of H_2_O_2_ balance enzymes was changed by osmotic stress. APX activity in the roots of PEG-subjected seedlings was 1.6–1.9-fold higher than that in the control. The maximum APX activity occurred at 1–3 h of stress influence (Figure 3a). CAT activity increased 2−6-fold during 4 h of PEG exposure. The peak of CAT activity in roots took place at 3 h of stress impact (Figure 3b). The roots of (SNP/+NO)-pretreated seedlings were characterized by the reduction in the stress-induced activation of APX and CAT relative to NO-untreated samples, which were still higher than the control. (SNP/+NO)-pretreated samples exposed to PEG for 4 h differed from the control by a 21–29% increase in APX activity and a 2.5- to 3-fold increase in CAT activity. In contrast, (SNP/−NO) pretreatment had no effect on APX and CAT activity in wheat roots under normal and stressful conditions (Figure 3).

### 2.3. ROS Content and Antioxidant Enzymes Activity after 24 h of 12% PEG Exposure

Control and (SNP/+NO)- and (SNP/−NO)-pretreated wheat roots 24 h later after seedlings’ treatment did not differ significantly in the O_2_^•−^ and H_2_O_2_ content under non-stressful conditions (Table 1). Exposure to 12% PEG for 24 h resulted in the enhancement of O_2_^•−^ production in wheat roots by 29% as compared to control. (SNP/+NO) pretreatment almost completely prevented PEG-induced O_2_^•−^ accumulation in the roots of seedlings subjected to stress during 24 h. The H_2_O_2_ content in the roots of SNP-untreated and (SNP/-NO)-pretreated plants increased more than 1.5-fold over the control under 24 h of stress exposure. The (SNP/+NO) pretreatment markedly reduced but did not prevent stress-induced H_2_O_2_ production. It was found that in the roots of (SNP/+NO)-pretreated seedlings exposed to PEG for 24 h, the H_2_O_2_ production was higher by 13.4% than in the control plants and lower by about 27% relative to NO-untreated and stress-subjected samples.

An increase in SOD and CAT activity by approximately 12% over the control was detected in the roots 24 h later after (SNP/+NO) pretreatment, while no significant changes in APX activity were detected. The stress exposure for 24 h caused a 2.5-fold increase in SOD activity, 1.95-fold increase in CAT activity, and the enhancement of APX activity by only 31% as compared to control. The roots of (SNP/+NO)-pretreated seedlings, in contrast to untreated ones, were characterized by the additional PEG-induced stimulation of SOD, APX, and CAT activities under 24 h of stress exposure. (SNP/−NO) pretreatment showed no effects of the active SNP on the studied antioxidant enzymes under control and stressful conditions.

### 2.4. Proline Accumulation in the Growth Medium

The amino acid proline belongs to low-molecular-weight osmolytes, and it is actively synthesized by plants in the response to water deficit conditions. It is characterized by a wide range of protective functions, including osmoprotective and antioxidant activity. Taking into account that exogenous pretreatment with active SNP reduced the level of oxidative stress in PEG-exposed wheat seedlings, it can be expected that Pro is an important component of NO-induced defence reactions. Therefore, we analysed proline excretion by wheat roots, which was determined by its accumulation in the growth medium of 7-day-old seedlings pretreated with (SNP/+NO) or (SNP/−NO) for 24 h and subjected to 12% PEG for 24 h.

**Figure 4:**
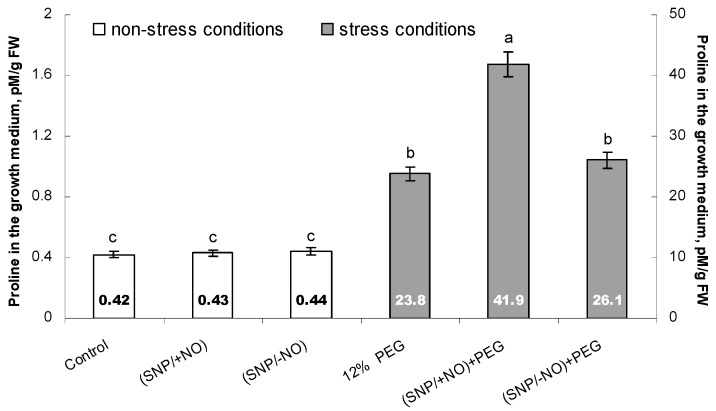
Effect of 2 × 10^−4^ M SNP pretreatment on proline (Pro) accumulation in the growth medium of wheat seedlings subjected to long-term (24 h) osmotic stress, caused by 12% PEG exposure. Data are given as mean values and their standard errors from three experimental and four analytical repeats. Distinct letters imply that the values differ significantly at *p* < 0.05 (ANOVA, LSD test).

No significant changes in Pro content were found under the influence of SNP; its concentrations in the growth medium of (SNP/+NO)-pretreated, (SNP/−NO)-pretreated, and control samples were approximately the same and ranged from 0.42 to 0.44 pM/mg (Figure 4). Osmotic stress tremendously increased Pro production by roots, causing more than 50-fold enhancement of its excretion to the growth medium, where its concentrations reached about 23.8 pM/mg. (SNP/+NO) pretreatment additionally stimulated stress-induced Pro production by roots, increasing its medium concentration by about 1.8-fold relative to SNP-untreated and stress-subjected samples, while (SNP/−NO) pretreatment had no such an effect. The level of Pro accumulation in the growth medium of (SNP/−NO)-pretreated and stress-subjected plants was the same as under 12% PEG exposure only.

### 2.5. The Levels of Malondialdehyde Accumulation and Electrolytes Leakage

**Figure 5:**
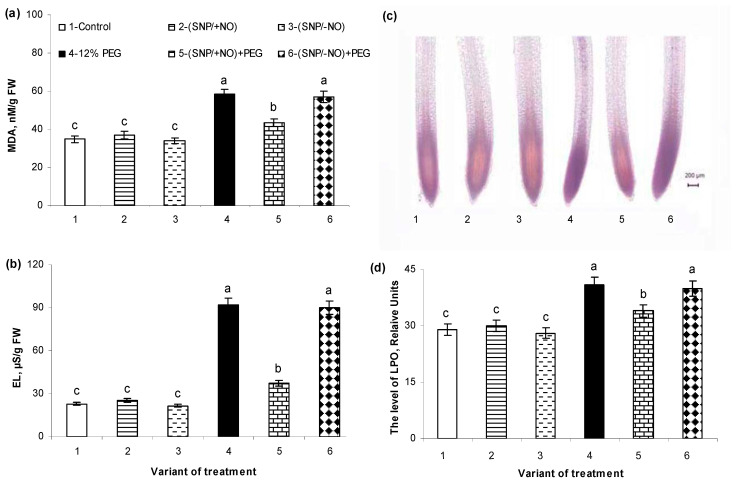
Effect of 2 × 10^−4^ M SNP pretreatment on cell structures integrity in roots of wheat seedlings subjected to long-term (24 h) osmotic stress, caused by PEG exposure. Accumulation of malondialdehyde (MDA) (**a**), level of electrolyte leakage (EL) (**b**), histochemical detection of lipid peroxidation (**c**), relative quantification of the lipid peroxidation (**d**). Image J software (NIH, Bethesda, MD, USA) was used to calculate relative level of peroxidation of lipids by integrated density. Data are the mean ± SE (*n* = 10). Distinct letters above the columns imply that the values differ significantly at *p* < 0.05 (ANOVA, LSD test).

Malondialdehyde (MDA) and electrolyte leakage (EL) were measured for the determination of lipid peroxidation (LPO) level and membrane structures integrity. Pretreatment with (SNP/+NO) or (SNP/−NO) had no significant effect on any of these parameters in the roots of 7-day-old non-stressed seedlings as compared to control (Figure 5a,b). Osmotic stress (PEG, 12%) during 24 h caused severe damage to membrane structures, which was evidenced by a 68% increase in MDA content and a 4-fold increase in EL level over the control. Exogenous (SNP/+NO) application had a protective effect on membrane structures of the roots of wheat seedlings under osmotic stress. (SNP/+NO)-pretreated samples were characterized by a decrease in stress-induced MDA accumulation and EL level, which exceeded control values by no more than 25% and 62%, respectively. The exhausted NO-donor had no effect on MDA content and EL level in the wheat roots under stress conditions. No significant differences in these two parameters were observed between samples pretreated or untreated with (SNP/−NO) and subjected to 12% PEG (Figure 5a,b).

### 2.6. Histochemical Detection of Lipid Peroxidation

Quantitative data regarding MDA accumulation and EL levels were confirmed during the histochemical studies using light microscopy and Schiff’s reagent, which stains aldehydes produced by lipid peroxidation (LPO) (Figure 5c). Histochemical analysis revealed that LPO predominantly localized in the root tips and central cylinder. The baseline level of LPO was detected in the tissues of control roots. Their tips, which are the zones of active proliferation and cell expansion, as well as the central cylinder, were stained by a purple colour (Figure 5c). Wheat exposure to 12% PEG for 24 h significantly increased the intensity of staining of all plant tissues, indicating the stress-triggered enhancement of LPO in roots in addition to the physiological one. (SNP/+NO) pretreatment decreased lipid peroxidation in the roots of wheat seedlings under osmotic stress as compared with SNP-untreated and stressed plants, while (SNP/−NO) had no such an effect (Figure 5c).

Ten representative images were then quantified using Image J software to identify differences in the staining intensity in root tissues of wheat seedlings pretreated or untreated with (SNP/+NO) and subjected to osmotic stress (Figure 5d). The intensity and area of staining were almost identical in the roots of control and (SNP/+NO)- or (SNP/−NO)-treated wheat roots under non-stress conditions. Under 12% PEG exposure the LPO level increased by 35% over the control. (SNP/+NO)-pretreated samples differed from NO-untreated ones by a significantly lower degree of stress-induced LPO, which exceeded the control values by no more than 11%. Under osmotic stress, (SNP/−NO)-pretreated samples were characterized by practically the same LPO level as SNP-untreated ones (Figure 5d).

### 2.7. The Percent of Cell Death and MI of Meristematic Cells of Wheat Roots

**Figure 6:**
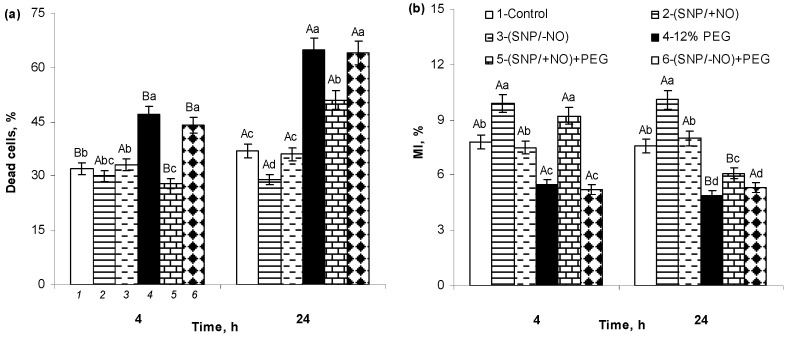
Effect of 2 × 10^−4^ M SNP pretreatment on the cells death percent (**a**) and mitotic index (MI) of meristematic cells (**b**) in roots of wheat seedlings subjected to short-term (4 h) and long-term (24 h) osmotic stress, caused by 12% PEG exposure. The numbers at the bottom of the columns indicate the variant of treatment. The number of roots in cell death assay: *n* = 10; the cells number in MI assay: *n* = 2000. Distinct lowercase letters above the columns indicate significant differences between variants of treatment at the certain time point at *p* < 0.05 (ANOVA, LSD test). Capital letters indicate significant differences for the same treatment at distinct time points at *p* < 0.05 (ANOVA, LSD test).

The level of cell death in roots of (SNP/−NO)-pretreated seedlings was not significantly different from control under normal growth conditions (Figure 6a). (SNP/+NO) application reduced the cell death rate by about 8% and 20% relative to the control 4 h and 24 h after of SNP pretreatment, respectively. Osmotic stress had a strong negative effect on the cell survival, increasing their death rate by 44% and 80% over the control after 4 h and 24 h of stress exposure, respectively. An active NO donor increased the viability of wheat cells under stress conditions. (SNP/+NO) pretreatment prevented PEG-induced cell death in roots at 4 h of stress exposure and decreased its level by about 22% at 24 h in comparison to SNP-untreated and stress-subjected samples. (SNP/−NO) pretreatment caused no changes in the stress-induced cell death rate.

An important indicator of the cells’ vital activity is the process of their division, which can be assessed using light microscopy by determining in the root apical meristem the parameter of mitotic index (MI), expressed as the percentage of dividing cells to the total number of analysed cells. MI ranged from 7.6 to 7.8% in the roots of 7-day-old control seedlings (Figure 6b). (SNP/+NO) pretreatment stimulated cell division as indicated by a 2.5% increase in MI over the control. Osmotic stress decreased the values of MI by 2.1% and 2.9% at 4 h and 24 h of PEG exposure, respectively, as compared to control. (SNP/+NO) pretreatment prevented the reduction in MI caused by 4 h of stress influence and significantly mitigated its inhibitory effect at 24 h of PEG exposure. Moreover, at the initial stages of stress, the (SNP/+NO)-stimulating effect on MI was still maintained in the root apical meristem, while exhausted SNP solution had no effect on the mitotic activity of the root’s cells either under non-challenging or stressful conditions.

### 2.8. Correlation Matrices

**Figure 7:**
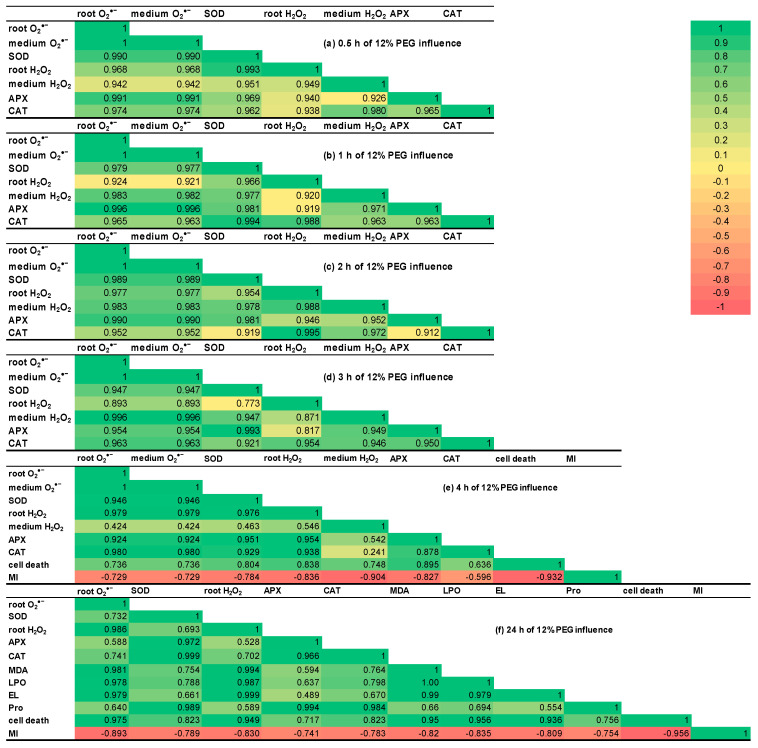
The correlation coefficient matrices for all examined parameters for 6–7-day-old wheat seedlings subjected to SNP pretreatment and osmotic stress, caused by 12% PEG. The matrices are coloured by magnitude and parity (green positive and red negative) for correlations at *p* < 0.05 significance level.

The analysis of correlation matrices revealed that during 12% PEG exposure for 0.5–2 h, almost all the studied indicators had a positive correlation with coefficients more than 0.9 (Figure 7a–c).

During the third hour of stress, the indicators could be grouped into two positive correlation clusters, with coefficients more than 0.9 and 0.773–0.893 (Figure 7d). The parameter of H_2_O_2_ in wheat roots showed declined correlation with APX activity, H_2_O_2_ in the growth medium, and O_2_^•−^ levels both in roots and in the medium. The parameter of H_2_O_2_ in roots had the weakest correlation with SOD activity during 3 h of stress exposure of wheat seedlings. H_2_O_2_ level in the roots demonstrated the reduction in its correlation with O_2_^•−^ generation and utilization.

The correlation coefficients between H_2_O_2_ accumulation in the growth medium and H_2_O_2_ content and SOD activity in wheat roots as well as O_2_^•−^ levels both in roots and in the medium decreased about two-fold during 4 h of PEG exposure (Figure 7e). After 4 h of stress influence, the correlation coefficients between the percent of root cell death and the indicators, illustrating redox status, decreased and reached the values of 0.636–0.895. CAT activity showed the weakest correlation with the percent of dead cells of wheat roots, while APX activity demonstrated the highest correlation with this parameter. Such data indicate the time-varying involvement of studied antioxidant enzymes in the processes of plant survival under osmotic stress. During the stress exposure of wheat seedlings for 4 h and 24 h, the parameter of MI showed a negative correlation with all other investigated indicators (Figure 7e,f). These data point to a switch from active growth to stress adaptation under PEG exposure. It should be noted that under normal conditions, active growth and adaptation processes are also antagonistic.

After 24 h of stress exposure, the most investigated parameters had changeable but positive correlation coefficients, except MI (Figure 7f). The levels of O_2_^•−^ and H_2_O_2_ in roots had a significant correlation not only between each other but also with MDA, EL, the percent of cell death, the level of LPO. The correlation between activities of antioxidant enzymes and MDA, EL, the percent of cell death, and the level of LPO declined and reached the less positive values 0.498–0.823. The correlation analysis data additionally confirm the stress-induced oxidative burst and its negative consequences on the plant cells. Pro in the growth medium has significant correlation coefficients with SOD, APX, and CAT activities. The correlations between Pro in the growth medium and ROS generation, MDA, EL, LPO level, and the percent of cell death were weaker (0.554–0.694) as compared to Pro’s relationship with the activity of the redox enzymes, indicating that Pro may act as an antioxidant.

## 3. Discussion

NO is an endogenous signalling molecule with multiple functions in the regulation of the plant’s development and its stress responses. Abiotic stresses have a negative impact on the water regime of plants, leading to osmotic and oxidative stress. This, in turn, disrupts the metabolic activity of the cells, limiting the growth and productivity of crop plants, including wheat, the world’s major crop. [15,21]. The root system of the plants is in direct contact with osmotic stress-inducing agents, and it determines the viability of the whole plant. NO plays a pivotal role in the formation of root architecture, modulating the growth of the primary roots, lateral and adventitious roots, and root hair development [22,23,24,25]. NO biosynthesis is increased in roots under abiotic stress conditions, and this can have a dual effect depending on the production level. NO can act as a signalling molecule at low concentrations, or it can be toxic at high concentrations and can provoke nitro-oxidative stress [24,25]. There is considerable evidence demonstrating the alleviation of the negative effects of different stresses via the exogenous treatment of plants with NO donors in proper concentrations through the root system, indicating their potential practical application to improve crop growth and productivity [15,21]. It has been previously shown that SNP pretreatment has stimulatory and protective effects on the growth of shoots and roots of wheat seedlings subjected to salinity or PEG-induced dehydration [15,26]. The beneficial effects of NO treatment were associated with its influence on the hormonal system, water regime, and also with the gene expression of dehydrins, which are well-known plant protective proteins [26]. In this study, we investigated the effect of the 24 h pretreatment of 5-day-old wheat seedlings (*Triticum aestivum* L.) through the root system with the active NO-donor (SNP/+NO) on various parameters of the redox homeostasis, as well as on the production of proline, the integrity of the membrane structures, and the mitotic activity and viability of the root cells under PEG-induced osmotic stress. Experiments were conducted using an exhausted SNP solution, (SNP/−NO), which served as an additional control of particular effects of NO.

The analysis of ROS content in the roots and in the growth medium revealed that the wheat seedlings produce O_2_^●−^ and H_2_O_2_ under control conditions at the basic levels, which were maintained at about the same values during the whole experiment (Figure 1a,b and Figure 2a,b; Table 1). In addition, the roots of the control plants excreted proline (Figure 4). These data are quite understandable, since it is well-known that the plant root systems secrete various biologically active compounds into the medium: sugars, organic acids, amino acids and fatty acids, sterols, enzymes, flavonoids, growth regulators, inorganic ions, RNS including NO, ROS, etc. [1,18,27]. Roots’ exudates have a wide physiological significance. For example, NO and ROS can be involved in the plants’ interactions with each other or with symbiotic microorganisms, as well as in the regulation of water and nutrient uptake. ROS production during normal metabolic processes occurs constantly. They are generated in cell walls and membrane structures, including plasmalemma, chloroplasts, mitochondria, peroxisomes, and in other cellular compartments, during 1-, 2-, or 3-electron reactions of oxygen reduction and in photoinduced reactions, and they are involved in the regulation of various metabolic processes [20,28,29]. In particular, H_2_O_2_ is capable of oxidating the thiol groups in protein molecules of enzymes or transcription factors, modulating the rate of the biochemical reactions or the expression of the corresponding genes. Apoplastic H_2_O_2_, hydroxyl, and superoxide radicals participate in the mediation of important plant processes, such as root cell elongation controlled by indole-3-acetic acid [30,31]. The baseline level of ROS production depends on the plant developmental stage, circadian clock, environmental and physiological conditions, and interactions with its root and leaf microbiomes, and it is under the influence of phytohormones, nutrient and water availability [1,32].

PEG-induced osmotic stress caused a rapid and reversible increase in root O_2_^●−^ and H_2_O_2_ production, as indicated by changes in their contents in the wheat tissues and growing medium (Figure 1a,b and Figure 2a,b). These results are consistent with the literature data. An increase in ROS production has been recorded in the different plant species exposed to adverse factors, inducing water stress in a huge number of studies [4,6,33,34,35,36]. Under such conditions, they can be both markers of the stress state of plants and signalling mediators in the adaptive reactions [9]. ROS can serve as short distance signals in plant tissues, acting along membranes between different organelles and/or neighbouring cells [32]. It is known that apoplast and cytoplasm interconnection plasmodesmata are involved in transducing RBOH-mediated cell-to-cell ROS and redox signals in plants [36]. This process is autopropagating and capable of transferring stress-induced ROS and redox signals from cell to cell over long distances [32]. The PEG-induced production of O_2_^●−^ and H_2_O_2_ led to the oxidative stress in the roots of wheat seedlings, as indicated by data on the increasing levels of MDA and EL (Figure 5a,b), damage to the cell membrane structures, and the enhancement of LPO (Figure 5c,d). Moreover, stress treatment has a strong negative effect on the viability of the root cells and their division (Figure 6a,b). The enlargement of stress exposure from 4 to 24 h significantly increased the death rate of the cells, notably decreasing their mitotic activity (Figure 6a,b).

ROS neutralization and plant redox homeostasis are ensured by AOS involving multiple antioxidant enzymes, in particular, SOD, CAT, and APX [37]. A significant increase in SOD activity was detected in the roots after 30 min of PEG exposure and reached a maximum after 1 h of stress, which then gradually declined but was significantly higher than the control after 4 h of the experience (Figure 1c). Then, SOD activity increased again after 24 h of stress exposure, exceeding the control by approximately 2.5-fold (Table 1). In this case, there was a direct connection between the levels of O_2_^●−^ content both in the roots and medium and the change in SOD activity in roots (Figure 1a–c; Table 1). An increase in the H_2_O_2_ content in the roots and medium under osmotic stress was in parallel with the activation of APX and especially CAT (Figure 2, Table 1). It is known that drought stress can activate plant enzymes POD and CAT differentially [18]. Literature data noticed that APX has higher affinity for H_2_O_2_ and is located in more diverse subcellular compartments than CAT, which works only in plant’s peroxisomes [20]. It is necessary to take into account that plants respond to stress-triggered oxidative burst not only through the stimulation of antioxidant enzyme’s activities but also through the up-regulation of non-enzymatic antioxidants [27,29,38,39].

It seems promising that the exogenous application of NO donors, including SNP, could increase plant stress resistance and crop productivity [24,25]. However, SNP is an unstable compound decomposing with the release of iron and cyanides, which can have a toxic effect on the plants, negating the positive effects of NO [40]. Meanwhile, compared to other NO donors, the relatively low cost and beneficial effects of its exogenous application were convincingly demonstrated in different plant species. Pretreatment with active or exhausted SNP solutions at a concentration of 2 × 10^−4^ M did not lead to oxidative damage in the wheat roots under control conditions, as was indicated by data on the production of O_2_^●−^, H_2_O_2_ (Figure 1a,b and Figure 2a,b; Table 1), MDA (Figure 5a), EL level (Figure 5b), histochemical analysis of integrity of cell membrane structures, evaluation of LPO (Figure 5c,d), measurement of MI, and cell viability (Figure 6a,b). Moreover, pretreatment with an active NO donor (SNP/+NO) promoted wheat root growth, as indicated by MI data (Figure 6a), whereas an exhausted NO donor (SNP/−NO) had no such effect. These data are consistent with the previously obtained data on root growth stimulation in different plant species through treatment with SNP in optimal concentrations [15,26,41]. The incubation of the root tips of 3-day-old maize seedlings in the presence of various NO donors, including SNP, stimulated their elongation [41,42]. Wheat seeds’ pretreatment with SNP or its presence in the germination medium at the concentrations of 50–200 µM promoted the subsequent increase in the linear sizes of the shoots and roots of 4–7-day-old seedlings [15,26]. NO-deficient mutants of *Arabidopsis thaliana* showed the delayed elongation of primary roots and were characterized by a poorly developed root meristem with abnormal cell division pointing to the pivotal role of NO in the root development [25]. On the other hand, it has been reported that NO is able to suppress root growth, as demonstrated in studies with NO-overproducing mutants or with exogenous NO treatments, indicating its concentration-dependent role in the regulation of root growth [25]. The exogenous NO treatment of tomato or cucumber plants inhibited the growth of the primary roots, although the growth of the lateral and adventitious roots was stimulated. This was accompanied by an increase in the branching of their root systems [22,23,43].

(SNP/+NO) pretreatment decreased the stress-induced accumulation of O_2_^●−^ and H_2_O_2_ in the roots and in the medium both under short-term (4 h) (Figure 1a,b and Figure 2a,b) and long-term (24 h) PEG exposure (Table 1), indicating the mitigation of oxidative stress in the wheat seedlings. At the same time, analysing the activity of antioxidant enzymes, we found that (SNP/+NO) pretreatment decreased the PEG-induced activation of SOD, APX, and CAT in the first 0.5–4 h of stress influence (Figure 1c and Figure 3a,b), whereas (SNP/+NO) pretreatment, conversely, resulted in the additional increase in SOD, APX, and CAT activities in the samples subjected to prolonged (24 h) osmotic stress (Table 1). Usually, the decline in ROS level is associated with the activation of endogenous antioxidant enzymes [28,29,38,39]. For example, SNP treatment increased the activity of antioxidant enzymes and upregulated the expression of APX genes in wheat plants under heat stress [34]. Spraying the soybean plants with 100 μM SNP reduced the levels of H_2_O_2_ accumulation and LPO and contributed to the additional activation of SOD, CAT, and APX in leaves under PEG-induced drought [6]. However, the SNP treatment of crambe plants decreased not only O_2_^•−^, H_2_O_2_, and MDA accumulation but also SOD, CAT, glutathione reductase, and APX activities under water deficit conditions [44]. The decrease in PEG-induced O_2_^●−^ and H_2_O_2_ production by seedling roots under the influence of (SNP/+NO) during the initial periods of stress (Figure 1a,b and Figure 2a,b) may be due to direct ROS interaction with the NO, leading to NOOO^−^ formation, which in turn may affect the catalytic activity of antioxidant enzymes [27]. It was shown that NO-triggered peroxynitrite accumulation in *A. thaliana* differentially inhibited the mitochondrial Mg-SOD, peroxisomal Cu/Zn-SOD, and chloroplastic Fe-SOD, which was due to tyrosine nitration [45]. Furthermore, SNP-induced ROS accumulation might be limited by the inhibition of the NADPH oxidase activity [27].

After 24 h of PEG exposure, (SNP/+NO)-pretreated samples exhibited a significant decrease in stress-induced H_2_O_2_ production and an almost complete prevention of O_2_^●−^ production, which was accompanied by an additional increase in the stress-induced SOD, APX, and CAT activity (Table 1). Obviously, during the first hours of stress exposure, antioxidant enzymes were not so active in reducing ROS generation (Figure 1 and Figure 3) as later in stress exposure (Table 1). Plants have both short-term and long-term responses to water deficit [46], and the regulation of SOD, APX, and CAT activity may be the result of distinct factors acting simultaneously. There is evidence that the additional activation of antioxidant enzymes induced by NO donors in wheat plants under salinity requires the coordinated action of abscisic acid (ABA), H_2_S, and NO [29,47,48,49]. The obtained results can indicate that NO may be involved in the regulation of antioxidant enzymes activities depending on the duration of osmotic stress. O_2_^•−^ or H_2_O_2_ were quickly scavenged in tissues under the initial stage of osmotic stress, and the activity of redox enzymes may not be the main factor in ROS regulation during the first hours of stress exposure (Figure 1, Figure 2 and Figure 3). Meanwhile, at later periods of stress, the ROS neutralization in (SNP/+NO)-pretreated wheat plants can be mediated by the stimulation of antioxidant enzyme activity (Table 1).

Various adverse factors, associated with osmotic stress, induce the accumulation of Pro in plant tissues [21,28,44,50,51,52]. This heterocyclic amino acid, which is characterized by osmoprotective, chaperone, and antioxidant properties, significantly contributes to the preservation of cellular molecular structures under conditions of water stress [7,8]. We found that a 24 h exposure to osmotic stress caused a nearly 50-fold increase in proline content in the growth medium of wheat seedlings, indicating the very strong magnification of its excretion by roots (Figure 4). Pretreatment with an active or exhausted SNP solution had no significant effect on proline production by the roots under non-challenging growth conditions. Meanwhile, (SNP/+NO)-pretreated samples differed from NO-untreated or (SNP/−NO)-pretreated samples by an additional almost 2-fold increase in stress-induced proline excretion into the medium (Figure 4). Such a significant increase in the proline content in the seedlings’ surrounding medium can play an especially important role in maintaining the hydration layer and structural integrity of roots in juvenile developmental stages under water deficit and osmotic stress conditions. These results are in good agreement with the literature data. There is evidence that NO donors usually have no significant effect on proline synthesis by plants under non-stressful conditions [53,54]. Meanwhile, NO pretreatment can enhance proline synthesis under water stress, as has been shown in *Lycopersicon esculentum*, *Ginkgo biloba,* and *Glycine max* [6,53]. The constitutive expression of the mammalian neuronal NO synthase gene (nNOS) in *Oryza sativa* led to an increase in proline accumulation induced by drought or salinity [55]. There is a suggestion that NO-induced proline accumulation probably has a stress-specific character and depends on the stress intensity. Additional studies are required to establish the role of NO in the regulation of proline synthesis, as well as its participation in the NO-dependent protection of plants under osmotic stress [53,54].

High MDA level is a marker of lipid peroxidation and its measurement is widely used for the detection of oxidative stress in plants. The significant increase in MDA production and EL level was observed under PEG exposure, indicating the severe damage of membrane cell structures in roots (Figure 5a,b). Pretreatment with (SNP/+NO) or (SNP/−NO) had no significant influence on the levels of MDA and EL under normal growth conditions, confirming the absence of the destructive effect of an NO donor in the used concentrations. (SNP/+NO)-pretreated samples differed from (SNP/−NO)-pretreated and from NO-untreated samples through a reduction in stress-induced MDA accumulation and EL rate, suggesting the NO-mediated mitigation of oxidative stress in wheat seedlings and also confirming the antioxidant activity of NO.

The SNP-dependent decrease in the activity of antioxidant enzymes during the initial periods of osmotic stress, which was revealed in this study (Figure 1c and Figure 3a,b), did not lead to subsequent oxidative stress in the wheat seedlings. On the contrary, SNP pretreatment contributed to the protection of the cells from oxidative damages, as evidenced by data on MDA content and EL rate (Figure 5a,b), which is consistent with the literature [28,56]. The histochemical analysis of the integrity of cell membrane structures using Schiff’s reagent revealed areas of ROS accumulation in the tips and central cylinder of control roots (Figure 5c). These data could be evidence of ROS involvement in the regulation of physiological processes such as cell’s division and expansion, as well as the formation of xylem and phloem vessels. Pretreatment with active or exhausted SNPs had no effect on staining with the Schiff reagent in comparison with control, which is consistent with the results of MDA and EL quantification, confirming the absence of a negative effect of SNP on the cell structures (Figure 5a,b). The action of osmotic stress led to the intense staining of the root tips and central cylinder, indicating severe damage to the cellular structures in the roots of wheat seedlings. (SNP/+NO) pretreatment reduced the area and intensity of stained zones in the roots of stress-subjected seedlings, whereas (SNP/−NO) had no such effect. Thus, the data of histochemical analysis provide further evidence of NO participation in the regulation of antioxidant defence in plants.

It is known that an oxidative burst, triggered by environmental stressors, can affect fundamental processes such as the cell cycle, cell division, and expansion. It can also initiate signalling towards cell death [30]. Cellular transitions from phase-to-phase are regulated by complex mechanisms in which the interaction between cyclins (Cyc) and cyclin-dependent kinases (CDK) plays an important role [57]. Redox perturbations affect both the activities and transcript levels of CYCs and CDKs through a specific transcription factor, TEOSINTE BRANCHED1-CYCLOIDEA-PROLIFERATING CELL FACTOR1 (TCP) [58,59]. Under oxidative stress, the interaction between a TCP and its promoter might be inhibited due to the formation of covalently linked TCP dimers via disulfide bonds, which in turn affects cell division [58,59]. It has been found in this work that osmotic stress increased the rate of cell death in roots (Figure 6a) and decreased the percentage of dividing cells in the tips, as indicated by MI data (Figure 6b). This may lead to the inhibition of growth and can ultimately reduce the stress resistance and productivity of wheat plants.

It should be noted that cell death occurs during normal plant ontogenesis, which is also evidenced by our findings. A baseline level of cell death ranging from 33% to 40% was found in the control samples (Figure 6a). Programmed cell death is necessary for the selective elimination of redundant or damaged cells, which is essential for maintaining tissue homeostasis. At the same time, cell death plays an important role in various developmental processes, including the formation of xylem and phloem vessels, as well as parenchyma and mechanical tissues [60]. SNP-pretreated samples showed approximately the same percentage of cell death as the control plants under non-challenging conditions, and a reduction in its stress-induced levels, indicating the ability of NO to participate in the maintenance of cell viability (Figure 6a). The presence of 12% PEG in the growth medium significantly reduced the values of MI, suggesting the inhibition of mitotic activity in the wheat roots (Figure 6b). An active NO donor stimulated cell division in the roots, as evidenced by the increase in MI values at 4 h and 24 h after (SNP/+NO) pretreatment. It is worth noting that the stimulating effect of (SNP/+NO) was retained even under short-term 4 h stress exposure. Furthermore, under prolonged 24 h stress, (SNP/+NO) pretreatment noticeably mitigated the stress-induced inhibition of cell division (Figure 6b). Pretreatment with exhausted donor NO had no significant effect on cell death and the mitotic activity of roots either in non-challenging or stressful conditions (Figure 6a,b). The stimulating and protective effect of (SNP/+NO) on the cell division and mitotic activity of wheat roots may be due to its influence on the gene activity linked with the cell cycle. In particular, the SNP treatment of tomato root segments stimulated the expression of cyclin genes, *CYCD3;1* and *CYCA2;1* [43], which may play a role in the NO-mediated regulation of root growth under both normal and stressful conditions.

## 4. Materials and Methods

### 4.1. Plant Material and Treatments

Winter wheat (*Triticum aestivum* L.) of cultivar Scepter was used as the object of the study. The seeds were obtained from the Chishminsky Breeding Station in Bashkortostan, Russia. The seeds were surface-sterilized with 96% ethanol and thoroughly washed with tap water. Then they were sown on Petri dishes containing filter paper soaked in 10 mL of 10% Hoagland–Arnon solution and were grown under illumination of 200 mmol m^−2^s^−1^ at 16 h photoperiod and ambient temperature of 22–24 °C for 3 days. Twenty 3-day-old plants were transferred into glass jars, containing 25 mL of nutrient solution. Regular changing of the solution (every day) was sufficient. The effects of NO were analysed using sodium nitroprusside (SNP), a donor of NO, at the concentration of 2 × 10⁻^4^ M, which, as previously shown, has a stimulating and protective effect on young wheat seedlings [15,26]. The experiments were conducted with the parallel application of active (SNP/+NO) and exhausted (SNP/−NO) solution, which lacks the NO activity and thus can serve as an additional control of active SNP. (SNP/−NO) solution was obtained by illuminating the stock (SNP/+NO) solution for 1 day at elevated temperatures. (SNP/−NO) treatment did not stimulate plant growth, which means it contained nitric oxide in quantities insufficient for a physiological effect on plants. 

**Figure 8:**
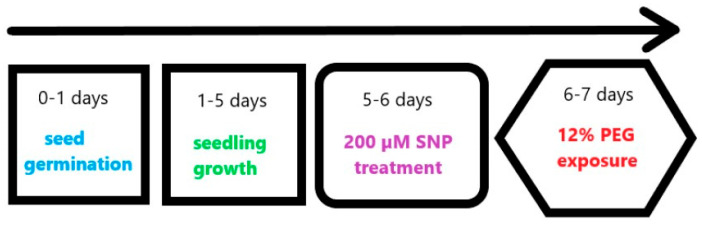
Schematic representation of the treatments in the study of the effects of SNP on wheat seedlings under osmotic stress (PEG, 12%).

The 5-day-old seedlings were SNP-pretreated through the roots for 24 h via supplementation with (SNP/+NO) or (SNP/−NO) into 10% Hoagland–Arnon growth medium. Control plants were grown on 10% Hoagland–Arnon solution. Then, the roots of wheat plants were carefully washed by distilled water, and the 6-day-old plant samples were subjected to osmotic stress through treatment with a mixture of 10% Hoagland–Arnon and 12% polyethylene glycol 6000 (PEG, PanReac AppliChem, Barcelona, Spain), which induce the physiological drought. The wheat plants were incubated during 1–4 h (short-term stress) or 24 h (long-term stress) (Figure 8). The roots of 6- or 7-day-old seedlings were used for the analysis of different parameters.

### 4.2. Assay of Antioxidant Enzymes

Superoxide dismutase (SOD, EC 1.15.1.1) activity was measured in the wells of a flat bottom plate for immunoassay (Costar, Washington, DC, USA) by spectrophotometer EnSpire 2300 (Perkin Elmer, Waltham, MA, USA) according to Beyer and Fridovich [61] at 540 nm. Enzyme extraction was performed by homogenizing the frozen roots of 5 wheat seedlings (250–300 mg) with extraction solution (0.15 M potassium phosphate buffer at pH 7.0) in a 1:10 ratio (*m*:*v*). The samples were then centrifuged for 10 min at 13,000× *g* and 4 °C. SOD activity was assayed by measuring its ability to inhibit the photochemical reduction of nitro blue tetrazolium (NBT, Merk, Darmstadt, Germany). For this purpose, 10 μL of the enzyme extract was mixed with 200 μL of reagent I (0.15 M potassium phosphate buffer (pH 7.8) containing 0.17 mM EGTA, 6 mM NBT, 3.3 mM phenazine metasulfate) and 10 μL of reagent II (Tris-EDTA buffer (pH 8.0) containing 1 mM NADH). The control was a mixture of 10 µL extraction buffer with 200 µL reagent I and 10 µL reagent II. One unit of the SOD activity (U) was defined as the amount of enzyme required to result in a 50% inhibition of the rate of NBT reduction at 540 nm. Results were given as a specific enzyme activity in Units/(mg protein × min).

Catalase (CAT, EC 1.11.1.6) activity was determined by measuring the decomposition of H_2_O_2_ [62]. The enzyme extracts were obtained by homogenizing the roots of 5 seedlings in 0.05 M potassium phosphate buffer (pH 6.2) in a ratio of 1:10 (*m*:*v*). The homogenates were centrifuged for 10 min at 13,000× *g* and 4 °C. The supernatants were used in the further analysis. The reaction was started by adding 0.15 mL 0.033% H_2_O_2_ to the 0.02 mL of enzyme extract in the wells of a flat bottom plate (Costar, USA). After 1 min, the reaction was terminated by adding 0.075 µL 4% (NH_4_)_6_Mo_7_O_24_ × 4H_2_O. CAT activity was measured by a Benchmark microplate reader (Bio-Rad, Hercules, CA, USA) as the change in the absorbance at 405 nm, defined as the destruction of complexes of H_2_O_2_ molecules with molybdenum ions. Results were given as a specific CAT activity in µM H_2_O_2_/(mg protein×min).

Ascorbate peroxidase (APX, EC: 1.11.1.11) activity was assayed as described by Verma and Dubey [63]. The roots of 5 seedlings were homogenized in extraction solution (50 µM K,Na-phosphate buffer (pH 7.8), 1% polyvinyl pyrrolidone (ABCR, Karlsruhe, Germany), 1 mM ascorbic acid) in a ratio of 1:3 (*m*:*v*) and incubated for 10 min at 4 °C. The extracts were centrifuged for 10 min at 13,000× *g* and 4 °C, and supernatants were used for the enzyme activity assay, which was conducted in the wells of a flat bottom plate (Costar, USA). The reaction was initiated by adding of 0.06% H_2_O_2_ to the mixture of 1.47 µL 50 mM K,Na-phosphate buffer with 15 µL 17 mM ascorbic acid, 15 µL 5 mM EDTA, and 5 µL extraction solution. H_2_O_2_ was not added to the control samples. The measurements were carried out for 90 s after the start of the reaction on the spectrophotometer EnSpire 2300 (Perkin Elmer, Waltham, MA, USA) at 290 nm. The APX activity was calculated using the following formula:

A = ((OD − OD_control_) × X)/(T × L × C), where

OD—optical density in experimental samples;
OD_control_—optical density of control samples;
T—time of reaction, min;
X—terminal dilution of extraction solution in the well;
L—layer thickness, cm;
C—protein content in the probe, mg.

Results were given as a specific activity in µM ascorbate/(mg protein×min). Total protein content in the extracts was quantified by the Bradford method [64].

### 4.3. Determination of Superoxide Radical in the Growth Medium

The extracellular production of O_2_^•−^ was measured by the oxidation of epinephrine (ICN, Costa Mesa, CA, USA) to adrenochrome [65]. A total of 1 mL of 10^−3^ M adrenaline solution was added to the water with the roots of ten intact control or experimental seedlings, which have been pre-incubated in glass jars in 0.025 mM CaCl_2_ solution for 1 h at 30 °C. The reaction of adrenaline conversion to adrenochrome was stopped by 0.05 n HCl followed by incubation for 15 min. The OD of the solutions was measured by a spectrophotometer Smart Spec Plus (Bio-Rad, Hercules, CA, USA) at 490 nm and expressed as nM O_2_^•−^/(g FW × min).

### 4.4. Estimation of O_2_^•−^ Content in the Roots of Wheat Seedlings

The level of O_2_^•−^ in root tissues was determined based on the method proposed by Chaitanya and Naithani [66]. The roots of 4 seedlings were homogenized in 0.1 M sodium phosphate buffer at pH 7.2, containing 1 mM sodium diethyldithiocarbamate to inhibit SOD activity, and the extracts were centrifuged at 13,000× *g* for 20 min at 4 °C. The supernatant (600 µL) was added to 1.4 mL of a reaction medium, containing 0.1 M sodium phosphate buffer (pH 7.2), 1 mM sodium diethyldithiocarbamate, and 0.25 mM NBT (Merk, Germany). The superoxide anion concentration was measured by its ability to reduce NBT. The absorbance of the reaction mixture was measured after 1 min at 540 nm using a spectrophotometer Smart Spec Plus (Bio-Rad, Hercules, CA, USA). The results were given as OD_540_/(g FW × min).

### 4.5. Extracellular H_2_O_2_ Content 

The content of H_2_O_2_ in the growth medium was determined by oxidation OPD with hydrogen peroxide according to Khairullin et al. [67]. Six-day-old seedlings were transferred to Petri dishes (ten seedlings per dish) with 0.01 M KCl solution (10 mL) 0.05% OPD. The reaction was initiated by the addition of 1 mL 0.025 M oxalic acid; the reaction mixture was stirred gently, and 0.2 mL aliquots were taken at 2 min intervals. The aliquots were applied to the wells of a flat bottom plate (Costar, USA) preliminarily filled with 0.05 mL 4 M H_2_SO_4_. The absorption of the samples was measured by a Benchmark microplate reader (Bio-Rad, Hercules, CA, USA) at 492 nm. The OPD oxidizing activity was expressed in absorption Units/(g FW × s).

### 4.6. Estimation of H_2_O_2_ Content in the Roots of Wheat Seedlings

H_2_O_2_ content was determined in the root extracts using the method described by Bellincampi et al. [68]. The roots of 5 seedlings (250–300 mg) were homogenized in 0.01 M potassium phosphate buffer (pH 6.0) in the ratio 1:5 (*m*:*v*) and incubated for 10–15 min at 4 °C. The extracts were centrifuged at 10,000× *g* for 10 min at 4 °C. One ml of the solution, containing 25 mM FeSO_4_ and 25 mM (NH_4_)_2_SO_4_ dissolved in 2.5 M H_2_SO_4_, was mixed with 100 mL 125 μM xylenol orange (Merk, Germany) and 100 mM sorbitol. The resulting mixture (100 μL) was used to initiate the reaction by adding supernatant (50 μL). H_2_O_2_ content was monitored with dye xylenol orange; when hydroperoxides are reduced by ferrous ions in acid solution, they form a ferric product–xylenol orange complex. The reaction was incubated in the dark at 25 °C for 1 h and absorbance were recorded by a spectrophotometer EnSpire 2300 (Perkin Elmer, Waltham, MA, USA) at 570 nm. H_2_O_2_ content was calculated using a standard curve based on the absorbance of H_2_O_2_ standards.

### 4.7. Malondialdehyde (MDA) Accumulation 

MDA was determined by measuring by its reaction with the thiobarbituric acid in accordance with Heath and Packer [69] with some modification. The frozen wheat roots (250 mg) were homogenized with 3 volumes of ice cold 10% trichloroacetic acid (TCA) and centrifuged at 10,000× *g* for 15 min. An assay mixture containing 1 mL of the supernatant and 1 mL of 0.5% (*m*:*v*) 2-thiobarbituric acid in 20% (*m*:*v*) TCA was heated at 95 °C for 1 h and then rapidly cooled in the ice bath. The absorbance of supernatant was monitored at 532 nm and 600 nm using a spectrophotometer Smart Spec Plus (Bio-Rad, Hercules, CA, USA). MDA content was calculated using 155 mM^−1^cm^−1^ as a coefficient of absorbance.

### 4.8. Estimation of Electrolyte Leakage (EL)

EL is considered as the marker of membrane stability. The level of EL in roots of wheat seedlings was determined in accordance with Bari et al. 2019 [35]. After all treatments, the surface of the roots was rinsed with distilled water. Then, the plants were incubated in the glass jars containing 20 mL of distilled water with shaking at 130 rpm on the orbital shaker–incubator ES-20 (Biosan, Riga, Latvia) at 30 °C for 2 h. The electrical conductivity of the incubation solution was recorded by a portable multi-range conductivity/TDS meter HI 8633 (HANNA Instruments, Catania, Italy). Results were given as electrical conductivity µS/g FW.

### 4.9. Proline Accumulation in the Growth Medium

For the determination of proline in the plant growth medium, 1 mL of growth solution from 10 wheat plants was mixed with 1 mL of glacial acetic acid–ninhydrin reagent and 1 mL of glacial acetic acid in a test tube, and the mixture was incubated in a water bath for 1 h at 100 °C [70]. The absorbance of the cold reaction mixture was measured at 522 nm using a spectrophotometer Smart Spec Plus (Bio-Rad, Hercules, CA, USA). The proline was calculated by comparison to a standard curve of proline, and the results were expressed as pM Pro/FW [71].

### 4.10. Analysis of Root Cell Death

The percentage of dead cells was analysed by the Evans blue method [35]. The roots of 5 wheat seedlings were kept in 10 mL of 0.25% (*w*:*v*) Evans blue (Merck, Germany) water solution at room temperature for 20 min. The amount of dye that entered into the cells corresponds to the degree of damage to tissue cells. The roots were washed three times by distilled water with gentle shaking; the trapped Evans blue was released from the plant tissues by homogenizing in 80% ethanol, and then the coloured solution was centrifuged at 12,000 rpm for 10 min. The optical density was monitored at 600 nm using a spectrophotometer Smart Spec Plus (Bio-Rad, Hercules, CA, USA), and the results of cell death in roots were given as percentages of frozen and thawed roots.

### 4.11. Histochemical Detection of Lipid Peroxidation

The lipid peroxidation of the root tips was determined histochemically [72]. The freshly cut off root tips were stained in Schiff reagent (Merck, Germany) for 1 h in the dark at room temperature until a purple colour appeared and then washed in 0.5% K_2_S_3_O_5_ in 0.05 M HCl solution [73]. Each variant of the experiment included 10 seedlings. The red/purple colour, indicating the localization of aldehydes formed during peroxidation, was monitored using a Biozero BZ-8100E microscope (Keyence Co., Osaka, Japan) at a magnification of x4. Then, images were analysed with ImageJ software (NIH, Bethesda, MD, USA) based on the integrated density multiplied by stained area to obtain relative total stained values [74]. For a better presentation of results, relative levels of lipid peroxidation were divided by 10^10^.

### 4.12. Mitotic Index (MI) Analysis

MI was determined from changes in the activity of the cells division in the roots’ apical meristem [75]. The root tips of wheat seedlings were collected after treatments and fixed for 1 h at room temperature in Carnoy’s solution composed of ethanol and glacial acetic acid in the of 3:1. The fixed root tips were kept in 70% ethanol under −4 °C. The slides were prepared by maceration method using 5% pectinase (Merck, Germany) and 5% cellulase (Merck, Germany) and stained by acetocarmine dye. Two thousand cells from the root meristem were used for the analysis of mitosis. Slide evaluations were performed in a light Biozero BZ-8100E microscope (Keyence Co., Osaka, Japan) at a magnification of ×20. Each variant of experiment included not less than 20 seedlings. MI was calculated as percent of the dividing cells.

### 4.13. Statistical Analysis

All experiments were performed in triplicate, and the experimental data were subjected to a one-way analysis of variance (ANOVA) using the SPSS 19.0 software (SPSS Inc., Chicago, IL, USA). Significant differences between values were determined using the LSD test at *p* < 0.05. Data in the figures are presented as mean values and their standard errors (±SE).

## 5. Conclusions

More and more evidence appears to show that NO is involved in a diversity of plant functions. Much progress has been made in the identification and characterization of enzymes involved in the biosynthesis and metabolism of NO and its derivatives [34,76,77]. NO plays a crucial role in plant signalling under different environmental stresses [21,78]. Under stress conditions, NO has several ameliorative functions as a signalling molecule [77]. The data available and the results of recent work revealed that NO is interconnected with the plant redox system and it’s regulation in response to different stress factors [18,39,79]. Osmotic stress triggers the excessive production of ROS of wheat seedling roots at the beginning of stress influence followed by oxidative stress-induced damage, whereas the application of active SNP decreased O_2_^•−^, H_2_O_2_ generation, EL level, and MDA content. It is known that environmental stresses induce short-term and long-term responses in plants [80]. Root-born hydraulic and chemical signals under water deficit conditions consist of two separate phases [81]. To avert the cellular damages, plants tightly regulate ROS production via the recruitment of enzymatic and non-enzymatic antioxidants. The exogenous supply of active SNP before 12% PEG treatment differentially regulated the activities of redox enzymes SOD, APX, and CAT in wheat roots. This regulation depended on time of stress influence, suggesting an important role for SNP during response to osmotic stress. During the first 4 h of osmotic stress, the activity of antioxidant enzymes of SNP-pretreated wheat roots reduced, while after 24 h of 12% PEG exposure their activities increased as compared to SNP-pretreated stressed plants. Under 12% PEG treatment SNP-induced declined of ROS production, which then decreased the damages of oxidative stress were observed in wheat roots. The effects (SNP/+NO) pretreatment on antioxidant enzymes activity demonstrated the dual role of NO donor, as active SNP involved in regulation of ROS signalling and in oxidative stress protection in wheat plants under osmotic stress.

## Figures and Tables

**Table 1:** Effect of SNP pretreatment (2 × 10^−4^ M, 24 h) on the content of O_2_^•−^ and H_2_O_2_ and the activity of SOD, APX, and CAT in the roots of 7-day-old wheat seedlings subjected to osmotic stress (12% PEG) over 24 h.

Treatment	Root O_2_^•−^ Content, OD_540_/(g FW × min)	Root H_2_O_2_ Content, µM/g FW	SOD Activity, Units/(mg Protein × min)	APX Activity, µM Ascorbate/(mg Protein × min)	CAT Activity, µM H_2_O_2_/(mg Protein × min)
Control	0.34 ± 0.017 b	153.85 ± 7.60 c	95.9 ± 4.5 d	1.31 ± 0.06 c	121.8 ± 5.9 d
(SNP/+NO)	0.33 ±0.019 b	154.85 ± 7.31 c	107.7 ± 5.2 c	1.23 ± 0.05 c	140.2 ± 6.7 c
(SNP/−NO)	0.34 ± 0.017 b	150.00 ± 7.40 c	92.3 ± 4.1 d	1.25 ± 0.06 c	118.2 ± 5.1 d
12% PEG	0.44 ± 0.020 a	240.00 ± 11.12 a	236.2 ± 11.1 b	1.71 ± 0.08 b	273.0 ± 11.6 b
(SNP/+NO) + PEG	0.37 ± 0.016 b	175.00 ± 8.52 b	292.2 ± 13.9 a	2.14 ± 0.10 a	322.8 ± 14.4 a
(SNP/−NO) + PEG	0.42 ± 0.021 a	235.06 ± 11.56 a	240.0 ± 11.6 b	1.74 ± 0.08 b	268.0 ± 14.3 b

Values are the mean of three replicates ± SE (*n* = 12). Different letters show significant difference at *p* < 0.05.

## Data Availability

Data are contained within the article.
